# Addressing substance use and mental illness among Quinault Indian Nation adolescents and young adults: community perspectives on community and cultural connection

**DOI:** 10.1186/s13722-026-00650-w

**Published:** 2026-01-22

**Authors:** Claire B. Simon, Julia Mackaronis, Elizabeth J. Austin, Madeleine J. Bentley, Jacqueline Gill, Evelyne Kalama, Stevie Charley, Joseph J. Nelson, Kevin A. Hallgren, Emily C. Williams

**Affiliations:** 1 Department of Family Medicine, School of Medicine, University of Washington, 4311 11th Ave NE, Suite 210, Seattle, WA 98105-6367 USA; 2 Roger Saux Health Clinic, Quinault Indian Nation, Taholah, WA USA; 3 Department of Health Systems and Population Health, School of Public Health, University of Washington, Seattle, WA USA; 4 Quinault Indian Nation, Taholah, WA USA; 5 Department of Psychiatry and Behavioral Sciences, School of Medicine, University of Washington, Seattle, WA USA

**Keywords:** Substance use prevention, Adolescent and young adult, American Indian/Alaska native

## Abstract

**Background:**

American Indian/Alaska Native (AI/AN) communities persist and thrive through intergenerational strength and resilience; they also continue to face disproportionate impacts from harms related to substance use and mental illness. There is an urgent need for evidence-based, culturally responsive approaches to increase protective factors against substance use and mental illness in AI/AN communities. Positive reinforcement of community and cultural connection may be protective against substance use and mental illness in adolescents and young adults (AYA). We partnered with the Quinault Indian Nation (QIN) to explore perspectives on community and cultural connection and how best to provide positive reinforcement that rewards engagement in cultural activities in order to build resilience, decrease substance use, and improve overall mental health.

**Methods:**

This study was guided by community-based participatory principles, approved by the QIN business council, and supported by a community advisory board of QIN members. We conducted semi-structured, 30–60-minute interviews with 15 QIN community members (both AYA and adults/elders). Interviews asked about community connection as well as community needs and preferences to address substance use and mental illness among QIN AYA. Interview transcripts were double-coded by three qualitative researchers and analyzed using a combination of Rapid Assessment Process and thematic analysis.

**Results:**

Our sample included 6 AYA and 9 adults/elders from the QIN community. Five themes emerged: (1) community connection involves reflecting community values in daily life, (2) QIN AYA have few formal and informal pathways to learn cultural practices and traditions, (3) QIN AYA frequently face logistical and/or structural barriers when trying to access cultural activities, (4) shyness, low confidence, isolation, and competing interests may also hinder QIN AYA engagement in cultural activities, and (5) new approaches to fostering cultural connection are needed to promote wellbeing and prevent harms related to substance use and mental illness.

**Conclusions:**

Both AYA and adult QIN community members described substantial barriers that hinder AYA engagement in cultural activities which build cultural connection and may support substance use and mental illness prevention. Community members emphasized focusing on upstream factors to encourage community and cultural connection and prevent substance use and mental health problems in the future rather than focusing on providing positive reinforcement for engagement in specific activities.

**Supplementary Information:**

The online version contains supplementary material available at 10.1186/s13722-026-00650-w.

## Background

American Indian/Alaska Native (AI/AN) people continue to foster intergenerational strength and resilience from within their communities. AI/AN communities also continue to face disproportionate impacts from harms related to substance use and mental illness. Related to historical and present-day traumas (e.g., colonization, dislocation, and structural racism), AI/AN populations have the highest prevalence of opioid overdose [1, 2] and death by suicide [3, 4] of any racial or ethnic group in the United States (US). There is an urgent need for evidence-based, culturally responsive approaches to increase protective factors against harms related to substance use and mental illness in AI/AN communities. In particular, prevention programs are needed for adolescents and young adults (AYA) who may be especially vulnerable to negative health outcomes associated with substance use and mental illness [5, 6]. Indigenous communities hold the environmental and cultural wisdom to address these health disparities, and programs that are co-designed to foster community and cultural connection among AI/AN AYA may act as a form of upstream prevention for harms related to substance use and mental illness [6, 7]. 

Fostering community and cultural connection in AI/AN AYA remain important challenges, particularly in the post-COVID era of social isolation and decreased in-person engagement. Providing positive reinforcement may encourage participation in activities that increase community and cultural connection. This manner of positive reinforcement is similar to contingency management, a behavioral intervention with a strong body of evidence in substance use treatment [8, 9], including in adolescent [10] and AI/AN [11, 12] populations. This form of intervention offers foundational theory on which to design a program that provides rewards for engaging in activities which promote cultural connection, with the goal of preventing or reducing harms related to substance use and mental illness.

In 2023, behavioral health providers from the Quinault Indian Nation (QIN) partnered with researchers at University of Washington (UW) to develop a program focused on providing positive incentives for cultural connection in QIN AYA as a means to reduce burden associated with substance use and other adverse mental health outcomes. To inform the design of this program, we conducted qualitative inquiry with AYA and older adult QIN community members to explore their perspectives on developing such a program for QIN AYA.

## Methods

### Study setting and population

The QIN is a sovereign nation whose reservation is located on the southwestern Olympic Peninsula, a remote area of Washington State. The QIN reservation includes over 200,000 acres of land including forest, rivers and 23 miles of Pacific Ocean coastline where members of the QIN community fish, hunt and gather on the same lands as their ancestors. QIN members are descendants of the Quinault, Queets, Quileute, Hoh, Chehalis, Chinook and Cowlitz people [13]. The Roger Saux Health Center (RSHC) is a federally qualified health center and includes behavioral health services located in Taholah, Washington, the headquarters for the QIN. This outpatient behavioral health clinic serves the population of the reservation, estimated to be around 1000 people, most of whom are enrolled QIN members.

### Community engagement

Consistent with community-based participatory research (CBPR) [14] principles, our research approach was a collaboration between behavioral health staff, community members, and researchers. All contributed to the research process equitably, and the goals of the research were driven by the needs and priorities of the QIN community. A community advisory board (CAB) guided all aspects of the study and was comprised of QIN community members who were either AYA or worked directly with AYA in the community. CAB members received compensation for their time and met with behavioral health staff and the research team monthly either in person or via video. In these meetings, the CAB collaborated to refine the aim of the study, iteratively develop an interview guide, identify and recruit interview participants, interpret results and disseminate findings to the community. As part of dissemination, CAB members, behavioral health staff and researchers hosted a large community dinner attended by an estimated 70 people where a CAB member shared findings and solicited feedback from the larger QIN community.

### Participant recruitment

We a priori planned to recruit 15 total interview participants based on funding and agreement with the QIN community. All QIN community members who were 13 years old or older were eligible to participate in interviews. Researchers partnered with behavioral health staff and CAB members to identify participants in the community who were AYA or caregivers of AYA, community members who were elders or had leadership roles in the community, or persons who worked closely with AYA (through schools, sports, behavioral health services, etc.). Behavioral health staff and CAB members contacted potential interview participants and provided information about the study. If interview participants expressed interest, they were then connected with the research team to schedule an interview. Snowball sampling was also used to identify additional eligible participants from the community after initial interviews were complete to reach our goal of 15 participants.

### Data collection

The interview guide was developed collaboratively by behavioral health staff, CAB members and the research team. Behavioral health staff and CAB members identified broad topic areas, and the research team drafted an initial interview guide. The interview guide was iteratively revised during a series of CAB meetings. The final guide was piloted with the first three interview participants before a final round of revision. Interview participants underwent an oral informed consent process and participated in a 30–60-minute semi-structured interview led by CBS in person or via video. Oral informed consent was obtained for all interview participants and parental consent was additionally obtained for interview participants who were under age 15. The interview asked open-ended questions about community and cultural connection, experiences of AYA in the community and preferences about a culturally- adapted incentivization program (with an initial focus on contingency management). Interviews were audio recorded and professionally transcribed. Interview participants received a $100 gift card after completion of the interview. All interview participants, including AYA, received the same compensation to honor their subject matter expertise.

###  Data analysis

Interview transcripts were analyzed using a combination of deductive and inductive approaches by three members of the research team, CBS, EJA, MJB. First, we used Rapid Assessment Process techniques where templated summaries were used to reduce data into neutral domains that aligned with interview questions [15–17]. Next we engaged in iterative coding by at least two independent coders and thematic analysis using team-based approaches rooted in grounded theory, including strategies of thick description (i.e., use of multiple participant quotes in all data summaries that presented themes and/or data interpretations), constant comparison (i.e., comparing each round of coding interpretations to the previous to continuously reflect and expand on interpretations and support dependability of themes throughout analysis), and use of qualitative coding memos (i.e., documentation of coder reflections (including biases) throughout the analytic process) [18, 19]. All coding was reviewed by the coding lead (EJA) for alignment and any discrepancies were reviewed by the full coding team and resolved via discussion using this constant comparison method or with consultation with the lead qualitative investigator (ECW). Full team consensus was reached on all coding and themes were iteratively reviewed by the lead qualitative investigator (ECW), full study team, and with behavioral health staff and the CAB. Final themes were verified with a larger community sample at the community dinner described above. During the community dinner, we conducted a form of participatory synthesized member checking, in which summarized interpretations of the data were presented back to a group participants and community members with similar lived experiences [20, 21]. The summarized interpretations were presented to the community by CAB members, so as to reduce the researcher-participant power dynamics that often result in traditional member checking approaches [22, 23]. During the community dinner, summarized themes were presented to community members (*n* = 70) who were given the opportunity to verify the accuracy of data interpretations and give feedback on whether interpretations were valid and trustworthy [20]. Feedback from community members were integrated into the final presentation of themes and confirmed by the full CAB and study team following the community dinner. This study was approved by the University of Washington Institutional Review Board and the Quinault Indian Nation Business Council (QIN does not have a formal IRB). All data is owned by the QIN, stored by the UW research team, and any secondary use of data requires CAB approval.

## Results

Among 15 QIN community member participants, 6 were 25 years or younger (AYA), 10 identified as female, and 13 were enrolled QIN members (see demographics in Table 1 below). Analyses identified 5 themes that characterized QIN community members’ perspectives on the current state of cultural connection, strategies to increase cultural connection, experiences of AYA in the community and preferences about manners of providing positive reinforcement (see summary in Table 2).

**Table 1:** Demographic characteristics of interview participants (*N* = 15)

Participant Characteristics of Sample (*n* = 15)	*N* (%^b^)
Age	
< 25	6 (40%)
25–54	8 (53%)
> 55	1 (7%)
Gender Identity	
Female	10 (67%)
Tribal affiliation	
Quinault	13 (87%)
Living situation	
On the reservation	10 (67%)
Primary mode of transportation	
Personal vehicle	11 (73%)
Driven by others	3 (20%)
Walking	1 (7%)
Parent of adolescent(s) or young adult(s)	
Yes	9 (60%)

**Table 2:** Summary of barriers and proposed solutions/needs to increase community and cultural connection among QIN AYA

Potential Barrier	Proposed Solution
**Individual level**	
• Low confidence to ask for help • Lost sense of purpose in adult community life​ • Boredom​ • Limited skills to promote mindfulness​ • Lack of motivation ​ • Competing interest in social media / phone use	• Increasing access to role models • Building individual skills for healthy relationships​ • Building individual skills that promote mindfulness & resiliency ​ • Learning traditional skills (e.g., regalia making, hunting & gathering)​ • Learning practical skills (e.g., personal finances)​ • Offering incentives for participating in program activities​
**Interpersonal level**	
• Busy caregivers • Fewer offerings of cultural activities • Less informal passing down / teaching of cultural traditions	• Involving caregivers and/or skills for parent-youth communication​ • Activities that connect young adults with elders​ • Having activities that connect youth to other youth (social, fun activities)​
**Community level**	
• Remote location (for adolescents, but also for program facilitators to access)​ • Changing community make-up (e.g., community getting more geographically spread out, more on/off reservation living) • Transportation barriers​ • Cost​	• Moving programs to different schools / locations​ • Ensuring program leaders have community knowledge / training • On and off reservations options • Providing transportation, whenever possible​ • Activities with other tribal communities​ • Free and/or very low cost​ activities

### Theme 1: Community connection involves reflecting community values in daily life

Participants of all ages identified community connection as an important component of wellness. Participants defined community connectedness as attending cultural events and showing up for other community members. One participant shared how AYA showing up for their peers is an example of how the community understands connectedness as being together and supporting others in times of need:

And I think it’s just being together. To me, I think that’s the biggest thing because they get to know each other, and they understand that, hey, this kid might not have as much as me, so I’m going to help them. You know, if there’s concessions, kids are like, “Hey, we’re gonna go to the concessions.” And, you know, the kid looks and shakes his head no, and they’re like, “Why?” And he kinda puts his head down. You know, they’re like, “Do you have money?” He’ll shake his head no. They’re like, “Come on, I’ll get you something.” And they look. “No, come on, let’s go. I’ll get you something.” You know? And that’s just how it is. That’s how it is in Taholah, is that we take care of one another. [P12, adult/elder].

Participants emphasized that showing up for other community members is especially important in such a small community like Taholah. Due to the tightknit nature of the community, doing something positive for someone would likely impact more than that one community member and have a positive effect on the QIN community overall:

*Because since it’s such a tightknit community and everybody knows everybody*,* a lot of the things one person can do can affect everybody. [P6*,* adult/elder]*.

Participants described the importance of community members beyond their immediate family showing up for AYA in formal (e.g., attending important community events with AYA) and informal (e.g., checking in with AYA after school) ways:

*My mom made me go to all of [the cultural events]. It was fun. I enjoyed it. And I got to learn a lot from it*,* met a lot of cool people. It was always a good experience. [P15*,* AYA]*.

*Sometimes if not a true auntie but a cousin or friend of the family who the kids call auntie. Anyway. It would be nice if there were family members or community members who would go to the school*,* see the kids who are acting out*,* and try to step in and lend a helping hand. Provide support. Somebody to talk to*,* anything. A lot of times kids will give respect to those people more than they would their own parents. [P2*,* adult/elder]*.

Teachers, coaches, cousins, and even parents of other AYA who were respected in the community could have a positive impact just by showing up:

*Even just them showing up to a game – the people who are working with our kids – showing up to a game*,* to show them like*,* “Hey*,* I’m here. Even outside of my work hours*,* I’m here supporting you. And I want you to know that I’m here and I want to be here.” You know what I mean? [P14*,* AYA]*.

Participants identified this as a cultural value that was previously strong in the community but had waned in recent years. They described this was in part due to many more respected adults, such as teachers and coaches, now live off the reservation which makes it harder for them to show up and support AYA outside of their regular work hours:

*The big difference is that teachers don’t live on the reservation. They live off the reservation now*,* compared to when I was growing up*,* the teachers lived on the reservation*,* and they were at football games*,* basketball games*,* name givings*,* birthdays*,* funerals*,* weddings*,* everything. You know*,* they were a big part of our community*,* and we don’t have that anymore. [P12*,* adult/elder]*.

Overall, participants reflected that community connection means living community values in everyday life in both formal and informal ways of interacting with one another.

### Theme 2: QIN AYA have few formal and informal pathways to learn cultural practices and traditions

Participants consistently stated that community and cultural connections were waning among AYA due to fewer opportunities to engage with culture, both formally and informally. Participants noted this was different than prior generations who had more opportunities to participate in programs that taught skills to engage in culturally important activities. Participants identified this as an unmet need for the current generation of AYA:

*When I was a kid*,* there was a lot of drum-making classes*,* necklace-making classes*,* beading classes*,* and stuff like that. I don’t see too many of those anymore. They’re not teaching the new kids our ways anymore. I think that’d help a lot if we started having more programs like that to teach them the things that we’ve always done. [P15*,* AYA]*.

Participants also highlighted the importance of fostering intergenerational relationships to pass on personal stories and cultivate a sense of identity and belonging through shared history. As one community member noted, fewer QIN AYA were learning these family histories:

*[Youth] don’t know their history. They don’t know of who’s related to who*,* how they’re connected*,* or anything that*,* and that’s what I’ve been working with my kids on. [personal story] […] But not very many kids can say that they know that family history piece*,* and it’s majorly needed. [P8*,* adult/elder]*.

Consequently, interview participants noted that AYA today have less knowledge of cultural activities and their significance than previous generations. Participants were concerned that QIN AYA are missing the foundational understanding of the history and significance behind cultural practices. Even when AYA engage in cultural activities, participants expressed concern that some AYA focus on tangible and material components of the practice rather than the cultural meaning and significance behind these activities:

*[…] but these kids – they don’t know what a button blanket is. They just think it’s something to make*,* and they want it to wear it. They want to drive through McDonald’s and get a button blanket. Well*,* no*,* a button blanket is each time you sew a button on*,* that’s a part of your life path*,* and each button represents what you’re wearing. [P9*,* adult/elder]*.

AYA participants expressed a desire to learn more about their cultural traditions and practices. One participant recalled enjoying engaging in cultural activities through a relative and wanting to become more involved:

*Like there’s been a few times my aunt has taken me to like jams and stuff*,* and I want to be more involved with stuff with that. Because I like to dance because the dances we do are fun*,* and the last time I went to a jam and we were dancing*,* it was really fun. And I kinda just want to be more involved with that and more involved in the stuff with my culture because I feel like I barely am. [P4*,* AYA]*.

Another AYA participant expressed a similar desire to learn more about their culture and highlighted the importance of passing down these traditions and practices to future generations:

*I think it’s cool because some people want to learn more about their tribes and stuff. Kind of like their languages and culture. […] That they can keep those kinda continuing. Because if it’s just left with the people who were gonna teach us*,* and then they don’t really get to teach anybody*,* then it kind of just dies off and we don’t have any more resources to kind of learn from it. [P13*,* AYA]*.

While older adult and elder participants were concerned that cultural practices and traditions as well as the history and significance of these traditions were not being passed down to the current generation of QIN AYA, AYA participants expressed a strong desire to engage in cultural practices and traditions. AYA, older adults, and elder participants all highlighted the importance of an intentional intergenerational connection in facilitating this knowledge sharing.

### Theme 3: QIN AYA frequently face logistical and/or structural barriers when trying to access cultural activities

While QIN AYA reported interest in strengthening their connections to culture, participants identified multiple barriers that may impact AYA’s engagement in cultural activities. AYA, older adults, and elder participants universally stressed the significance of transportation as a barrier to engaging in community and cultural events due to the remoteness of the QIN reservation. Community members noted that any program developed to promote community and cultural connection would need to provide transportation and chaperones as caregivers of AYA in the community might not have the capacity to provide this themselves:

*It [transportation] is a big barrier*,* especially for single-parent families*,* when they have to work*,* and the child needs to get to football practice*,* basketball practice*,* baseball practice. So*,* it’s all during the week*,* and they have to find ways to be able to make sure their kids get out there. So*,* it is a big barrier. [P7*,* adult/elder]*.

*We’re remote so traveling can be difficult. Some of them don’t have the kind of families that could make that happen for them. They would need chaperones and different people in the community to help with that. [P5*,* adult/elder]*.

In addition to transportation, older adult participants reported that caregivers of AYA often had multiple competing obligations that limit their capacity to support AYA in engaging in activities:

*We don’t have the interaction with kids that we used to have with the adults and the elders. And I don’t think that we have the parent involvement in school. [P12*,* adult/elder]*.

Adult participants emphasized that getting parental buy-in would be critical to the success of any program aimed at increasing community and cultural connection for QIN AYA:

*Transportation and parent buy-in. That’s the main thing. If the parents buy into it and understand that it’s something that’s gonna benefit and help their kid*,* and the communication is there*,* they’re more willing to encourage their kid through it. But the thing is that a lot of times*,* parents will just go and drop their kid off and take off not know what’s going on. Probably getting more involvement and understanding of what the program’s gonna be doing*,* what the program’s about*,* and how it’s gonna help their kids*,* I think they’d have buy-in in it and feed them. [P10*,* adult/elder]*.

Adult participants also noted cost as a barrier to AYA and their families participating in cultural activities. Many traditional QIN activities, such as clamming and button blanket making, require expensive tools and materials. Any program developed for AYA would need to cover the cost of any necessary tools or supplies to ensure all AYA can participate.

*If you need equipment or if you need anything in particular to participate*,* then money is going to be a problem. [P2*,* adult/elder]*.

Finally, some older adult participants noted that the changing demographics of the QIN community can make it more difficult to build cultural connections. Many QIN AYA now live off the reservation which can make it harder for them to stay connected. Participants also noted that many AYA in the QIN community have connections to other tribes, some of which have shared cultural traditions and practices while others that are further away may not. Participants shared that these changing demographics highlights the importance of intertribal connection in addition to connection within the QIN community.

*A lot of these kids aren’t just Quinault*,* either*,* but that they have family members from as far as Neah Bay*,* and all the way down as far as California*,* and all the way out as far as Montana*,* I believe. We have some kids that have family members all the way over there. So*,* not just being Quinault tribe*,* but also tribes in general*,* or even having that connection piece of where we do the same things as what’s done at Neah Bay or La Push or Hoh River. But having culture is so much needed*,* but it’s not just the fluff pieces or even to where it’s a small demonstration; but this needs to be a major program within the nation*,* because we’re struggling with the culture pieces with these kids. We’re struggling with the connection. [P8*,* adult/elder]*.

Overall, adult participants noted many barriers to cultural connection for QIN AYA. While some barriers were straightforward with clear solutions such as providing transportation, others were more complex and less straightforward to address. It should be noted that this theme did not emerge from AYA interview participants.

### Theme 4: Shyness, low confidence, isolation, and competing interests may also hinder QIN AYA engagement in cultural activities

In addition to structural barriers, participants perceived AYA as struggling to join or advocate for cultural activities due to multiple interpersonal barriers that they believed were exacerbated by a wider cultural shift away from in-person engagement. Participants shared that general stress has led community members, including AYA, to be more reserved and less engaged in the community than they had been previously:

*Like I think right now more than anything… I think there’s so much going on just in the world and in our little villages*,* and people are kind of more reserved. Some people don’t want to say anything*,* they don’t want to either participate because they don’t like somebody*,* or they just don’t feel like it’s gonna help. [P1*,* adult/elder]*

*Kids are lot different than when I was growing up.[P11*,* adult/elder]*.

Multiple community members noted that social isolation resulting from the COVID-19 pandemic as well as the rise of technology (e.g. social media, gaming) have negatively impacted QIN AYA’s connection to their community resulting in the current generation of QIN AYA being more withdrawn:

*Part of it is technology. The other part is*,* I don’t know if it’s from COVID. Like my son*,* he’s not a people person. He’ll do sports*,* but he’s just not a people person. [P11*,* adult/elder]*.

*I think what I reflect at watching – because I’m a people-watcher – what I see with the youth is they’re withdrawn. It’s a combination of COVID*,* because they just had it. They experienced it*,* too*,* and that they*,* they’re used to not being heard. [P9*,* adult/elder]*.

Participants noted that some AYA lacked the confidence or trust to ask family members and elders for support:

*A lot of adolescents don’t like to go to their elders and family members if they don’t feel comfortable enough talking to them.” [P4*,* AYA]*.

*“If you don’t have people to talk to and you’re kind of just quiet*,* don’t ask for help and stuff*,* it kinda just impacts you and makes it harder to kind of speak. [P13*,* AYA]*.

Finally, participants reiterated that QIN AYA were eager to learn, even if they lacked the confidence and interpersonal skills to engage at first. If a program were offered that gave AYA the opportunity to learn cultural practices, participants believed that QIN AYA would gravitate toward it:

*They wanna learn. It’s just that they’re scared to ask. And that’s the biggest problem with a lot of the youth is they’re scared to ask because maybe their family didn’t have time. Maybe someone didn’t have time to try and teach them*,* or they feel like they’re bothering someone while they’re trying to do something. So*,* they don’t ask. But when we offer something to them*,* they gravitate towards it. And so*,* they wanna do it. [P10*,* adult/elder]*.

In summary, participants highlighted the increased prevalence of shyness and isolation in the current generation of QIN AYA which they attributed to a shift away from in-person activities that build connection due to the COVID-19 pandemic and less in-person engagement.

### Theme 5: New approaches to fostering cultural connection are needed to promote general health and wellbeing as a means for improving substance use and mental health

Adult participants expressed the need for a program to promote health and wellbeing in QIN AYA that better met the needs of AYA today, in particular through encouraging participation in programs that incorporate practical life skills and cultural practices:

*[…]I know going through school*,* you’re not taught about being an adult. They’re not really being prepared for the world they’re going to venture into after high school*,* finances [P11*,* adult/elder]*.

*I don’t think that college is for everyone. I believe that some students*,* they already know that once they’re done with high school*,* that they’re done with schooling. And a lot of them*,* they’re looking at*,* “I’m going to be a fisherman*,* I’m going to be a crabber*,* I’m going to do this*,*” and you can utilize their treaty rights. And it’s fine. [P7*,* adult/elder]*.

Adult participants emphasized the abundant natural resources on the reservation, noting the opportunity for AYA to learn to use these resources would not only build cultural and community connection but also provide them with an opportunity to develop a career:

*I just think that they need to embrace what they have here. The resources in this area are plenty. There’s things they can do. I got with a couple of them*,* and they never been out picking Indian tea here. They don’t even know what to look for*,* for Indian tea. And then*,* they have an elder that’s up in Queets that’s actually started her own business for selling Indian tea*,* and everything*,* her own medicine for people. But yeah*,* it’s just getting them outside*,* turning off their videogame. [P10*,* adult/elder]*.

*It would be great if we had elders that would come and help set up a program based off of like*,* ‘These are things that you should look for. These are how to properly handle your weapons.’ I think they do actually have those*,* but I think it’s quarterly or something. But to be able to say how to handle your firearms*,* how to protect your firearms*,* how to actually hunt*,* what time of the season; to be able to break down the whole education part of it*,* to be able to show them like*,* ‘Hey*,* if you’re going elk-hunting*,* this is your time of the year. If you’re going bear season*,* this is your time of the year. And these are the things you need to look for if you’re going hunting*,* if you’re out there hiking around*,* and you’re going to go out by yourself. Make sure you have the proper safety equipment*,*’ all that stuff. I feel like if I had that*,* there’d be more youth that are willing to go out and hunt. [P7*,* adult/elder]*.

*And I think that our elders need to be there because I think our kids need to hear the stories. You know*,* let’s say that you say*,* “Hey*,* we’re gonna have a night where we are gonna be working on cedar baskets.” And if you have elders there*,* the elders are gonna talk about who they used to watch make cedar baskets. They’ll talk about*,* you know*,* ‘When I was little*,* such-and-such used to do all kinds of cedar baskets.’ And then they go into a big story. [P12*,* adult/elder]*.

Adult participants recommended leveraging cultural activities as way to build confidence and interpersonal skills via role modeling & relationship building as a way to prevent substance use and mental illness. A program that encouraged engaging in cultural activities was seen as an opportunity for AYA to overcome some of the interpersonal challenges facing the current generation of AYA and ultimately lead to improved overall health.

*I think it would be huge. The benefits would be connectedness and getting to know each other as a person*,* and being able to know them. [P1*,* adult/elder]*.

*Giving this new generation*,* the teenagers*,* a better aspect that*,* Hey*,* there’s a world out there that needs you and wants you. [P9*,* adult/elder]*.

Adult participants recommended new approaches to build confidence and interpersonal skills in AYA that incorporate practical life skills and cultural practices to promote overall health and wellbeing as a means for improving substance use and mental health. It should be noted that this theme emerged predominantly from interview with adult and elder community members.

## Discussion

In this study, UW researchers partnered with QIN behavioral health providers and QIN community members and conducted qualitative interviews to inform the design of a community-based program to reduce the harms of substance use and poor mental health for QIN AYA. In interviews, QIN community members discussed their perspectives on community connection and reflection of community values in daily life. They noted multiple community-level changes over time, as well as some structural and related interpersonal barriers that make it more challenging for AYA to build community and learn cultural practices and traditions than during previous generations. Overall, they described a strong need for new approaches to foster cultural connection to promote self-confidence and self-knowledge and ultimately health and wellbeing. While interview participants did not emphasize a need to reduce risk for harms related to substance use and mental health specifically, this remained a high priority for behavioral health and CAB partners.

Though we began with contingency management as a foundational potential intervention, with a focus on providing incentives for engagement in activities that may prevent substance use and/or mental health problems, interview participants did not demonstrate particular interest in this type of intervention. Instead of incentive driven interventions, we heard QIN community members were most interested in upstream interventions focused on building resilience through community and cultural connection. The strategy of building resilience through community and cultural connection described by participants reflects that, in AI/AN communities, culture is treatment and shares some similarities with Healing of the Canoe [24], a curriculum developed for Pacific Northwest tribal communities that teaches life skills as substance use disorder prevention [25] and Living in 2 Worlds program, a substance use prevention curriculum [26] for urban AI/AN middle school students which aims to strengthen resilience and cultural engagement. Other programs aimed at reducing substance use in other AI/AN communities have been developed and often focus on building cultural and community connection through specific traditional ceremonial practices such as drumming, sweat lodge and talking circles [27]. This manner of promoting engagement in cultural activities in order to promote health and wellbeing is consistent with a treatment conceptual model [25] of traditional and grassroots cultural behavioral interventions.

Interview participants highlighted many important barriers that AYA face that prevent them from engaging in activities that build community and cultural connection. These structural determinants likely reflect the long-term impacts of both historical and contemporary racism, colonization, and trauma on the daily lived experiences of QIN community members. Interview participants noted barriers present at the individual level (low confidence), which may be an outcome of disconnection, trauma, and digital displacement. Barriers were also noted at the interpersonal level (busy caregivers and less intergenerational knowledge transfer), potential outcomes of systemic inequities including long work hours, commuting, and historical disruption of family systems. Additional barriers were noted at the community level (remote location and transportation), due to residual impacts of colonization such as stolen land and resources. A community-based program could address these specific barriers by providing positive reinforcement to engage AYA who might have some hesitation to participate, engaging elders and other community members in the program to provide access to positive role models and opportunities to transfer intergenerational knowledge (theme 5) and ensuring that transportation is provided so all AYA are able to participate (theme 3) (see summary of barriers and potential solutions in Table 2 below).

This study has several important limitations. First, though several of our themes emerged from data collected from QIN participants of all ages (e.g., reflecting community values in daily life, few pathways to learn cultural practices and traditions, and shyness, low confidence, isolation, and competing interests hinder engagement in cultural activities) other themes emerged exclusively from interviews with adults and elders (e.g., barriers to cultural activities, new approaches to fostering cultural connection needed). Thus, perspectives of AYA are not reflected in all themes, and further work is needed to co-design specific interventions with AYA, explicitly. However, the learnings from adults and elders in the present study provide key foundational knowledge. Many of these adult and elder participants grew up in the community, which likely allows them to see clearly the barriers the current generation of AYA face to engaging in cultural and community connection relative to those in prior generations, while current AYA may not have this historical perspective and may have less insight into specific barriers they experience that result from changes in culture over time. Adults and elders who grew up in the community frequently described programming available during their AYA years that supported community and cultural connection that are no longer available to current AYA. Current AYA may not be aware that these programs previously existed and therefore may not recognize what they are missing. Second, while we conducted interviews with community members of different ages and with different roles in the community, this qualitative data may not generalize to all QIN community members. We a priori planned 15 interviews due to funding and team capacity, and this may have limited our ability to saturate themes. Third, our study explored QIN community perspectives and while other remote AI/AN communities may share some common perspectives about community and cultural connection, they may not experience the same barriers and may not have the same potential solutions to these barriers. Future studies should co-design community-based program to target upstream factors that may prevent substance use and mental health problems in QIN AYA and evaluate the implementation of this program.

## Conclusion

In this collaborative community-engaged study, QIN community members described substantial barriers AYA face to engaging in cultural activities that build cultural connection and may be health-promoting. Community member perspectives emphasized upstream factors – such as community values on health, intergenerational mentorship, and indigenous skill building – that may be important leverage points to prevent substance use and mental health problems in the future. This work also highlights the importance of community engagement throughout the research process; learnings from our work identified opportunities to develop a community-based program that directly addresses present barriers to community and cultural connection in QIN AYA and may be more responsive to the needs of community members.

## Supplementary Information

Below is the link to the electronic supplementary material.

Supplementary Material 1

## Data Availability

The datasets generated and/or analyzed during the current study are not publicly available due to tribal data sovereignty. Data are however available from the authors upon reasonable request and with permission of the community advisory board.

## Abbreviations

AI/AN: American Indian/Alaska Native
QIN: Quinault Indian Nation
AYA: Adolescent and Young Adult
CAB: Community Advisory Board
RSHC: Roger Saux Health Clinic
