# Supplementary material for: Addressing substance use and mental illness among Quinault Indian Nation adolescents and young adults: community perspectives on community and cultural connection
Source: Addict Sci Clin Pract. 2026 Jan 22;21:14. doi: 10.1186/s13722-026-00650-w (PMC12829219; doi:10.1186/s13722-026-00650-w)
Supplement: Supplementary file 1 — Supplementary Material 1 [file 13722_2026_650_MOESM1_ESM.docx]

PREAMBLE:

Thank you for speaking with me today. A group of us from UW and the behavioral health clinic, are working to develop a community program that engages adolescents in rewarding activities to help reduce risk for mental health and substance use problems. We want to hear from adolescents and those around them about interest in this type of program and how the program should work. We plan to share the general findings back to the community, and, if the community desires, implement the program.

We will record the interview, but your name will not be linked to the interview. We may use your responses and some short quotes to develop the program and for other research purposes. There are no right or wrong answers. You may choose to skip any question or end the interview at any time. The interview will take approximately 60 minutes and will receive a gift card after completion of the interview.

Do you consent to me recording our conversation? Do you have any questions for me before we begin?

(begin recording)

Identity/Connection to community

We are hoping to create a program for Quinault and Queets adolescents. What is your connection to this community?

What community/school programs for adolescents do you have experience with, if any?

- For example, Youth opportunities program (YOP), the teen center, sports, music, dance.

What do adolescents in your community like to do in their free time?

- For example, sports, crafts etc.
- If all barriers were removed what else would adolescents like to do?

What do healthy relationships with friends and family look like to you?

- How do you know if someone is in a healthy relationship?
- How should adolescents learn about healthy relationships?

What adults do adolescents feel the most comfortable with?

- Who in the community is a good role model?

Need for a program

What challenges do you see for mental health and substance use for adolescents?

- What do you think contributes to these challenges?

Do you think there is a need for more programs that provide positive reinforcement/community connection?

- Why do you think there is a need?
- What would be the benefit of a program that provides positive reinforcement?

Content of Program

What are important life skills for adolescents to learn?

- What is the best way to learn these skills?

What activities do you think would help adolescents feel more connected to their culture and community?

- What would be the benefits of adolescents participating in these activities?
- Would there be any risks?
- How do you think adolescents would feel about this program?
- How do you think the community would feel about this program?

Are there other positive activities that we should encourage adolescents and young adults to engage in?

- Why? What else?

Part of the goal of this activity is to reward adolescents for participating in structured activities. How should we reward adolescents for participating in the program?

- Many programs give folks cash or gift cards for participating in programs, how do you feel about that?
- How to you think others in the community would feel about giving rewards?
- What about providing food? What else would be a good reward?

Program Structure

Who should we invite to participate in the program?

- Are there certain groups of people who you think would especially benefit?
- For example, some community members have identified newly graduated seniors as a group who would especially benefit. What do you think about this?

Where/as part of what program in the community should the program take place?

- For example would you feel about it taking place in schools, in the mental health clinic, as part of the wellness court?
- Is there any other place that youth go where a program like this would make sense?

Do you think the program should take place on the reservation, off the reservation or in the city?

Who should lead the program?

- Should it be people from the community or outside the community?

What do you think could make it difficult for adolescents to participate in the program (such as lack of transportation etc)?

- What do you think would make it easier?

How can we make sure that the program is sustainable?

- How can we ensure the program is able to continue long term?

Demographics

I would like to ask a few general questions about you do that we know whose voices contributed to this program.

How old are you?

What is your gender?

What is your race and or ethnicity?

Do you have a tribal affiliation?

Do you live on or off the reservation? Where do you live?

What is your primary mode of transport?

Do you have children who are adolescents or young adults?

Do you have any general thoughts about this program or anything else you would like to share with us?

(stop recording)

CLOSING STATEMENT:

That is all the questions that I have for today. Do you have any questions or additional comments for me as we conclude this interview? We as a team thoroughly appreciate you making the time today to speak with us and participate in our project. If any questions come up after our session concludes today, please feel free to follow up with us via email. We plan to host a community dinner to share a summary of these interviews. Thank you again.
